# The genus *Ptilophora* (Lepidoptera, Notodontidae) in China, with description of a new species

**DOI:** 10.3897/zookeys.61.494

**Published:** 2010-10-11

**Authors:** Liusheng Chen, Guohua Huang, Min Wang

**Affiliations:** 1 Department of Forestry, College of Agriculture, Shihezi University, Shihezi, Xinjiang, 832000, China; 2 Institute of Entomology, College of Bio-safety Science and Technology, Hunan Agricultural University, Changsha 410128, Hunan Province, China; 3 Department of Entomology, South China Agricultural University, Guangzhou 510642, China

**Keywords:** Lepidoptera, Notodontidae, Ptilophora, new species

## Abstract

The genus Ptilophora Stephens in China is briefly reviewed, with the description of Ptilophora nanlingensis **sp. n.** The new species is most similar to Ptilophora horieaurea in wing pattern and to Ptilophora jezoensis in male genitalia, but they can be distinguished from each other by the following characters: forewing bright reddish brown in Ptilophora nanlingensis, chestnut brown in Ptilophora horieaurea; costa of male genitalia pointed, with a rounded subapical process ventrally in Ptilophora jezoensis, costa rounded, with apex inflated, and with pointed subapical process ventrally in Ptilophora nanlingensis. A key to the Ptilophora species from China and adjacent areas is presented and a distribution map is given. The holotype of the new species is deposited in the Department of Entomology, South China Agricultural University, P. R. China.

## Introduction

The notodontid genus Ptilophora was established by Stephens in 1828 with Phalaena variegata Villers, 1789 as its type species. Currently, the genus consists of six species that are distributed in Europe (except northern Europe and the Iberian Peninsula), Caucasus, Asia minor, China, Far East Russia, Korea and Japan (Sugi 1982; Schintlmeister 1984; Kobayashi 1994; Wang 1996; Park et al. 1999; Wu and Fang 2003; Schintlmeister 2008). Hitherto, three species of the genus are restricted to China, Ptilophora rufula from Taiwan, and Ptilophora ala and Ptilophora horieaurea from mainland China. Adults of Ptilophora species emerge in late autumn. Schintlmeister and Fang (2001) described Ptilophora jezoensis ala as a new subspecies of Ptilophora jezoensis in Shaanxi Province; later, Kishida and Kobayashi (2002) revised the genus Ptilophora, divided the species into two species-groups: plumigera group and jezoensis group, and described two new species belonging to the jezoensis group from South West China: Ptilophora horieaurea Kishida and Kobayashi, 2002 and Ptilophora fuscior Kishida & Kobayashi, 2002. Schintlmeister (2008) raised Ptilophora ala to species, based on comparing the types of Ptilophora ala with those of Ptilophora fuscior, and reduced Ptilophora fuscior to a junior synonym of Ptilophora ala. In this paper, a new species of Ptilophora is described from China.

## Key to the species of Ptilophora Stephens from China and adjacent areas

**Table:** 

1	Antenna black; forewing with a prominent discal spot; median fascia of hindwing blackish; male genitalia with rounded valve	2
–	Antenna brown to chestnut brown; forewings without prominent discal spot, median fascia of hindwing whitish or absent; male genitalia with irregular valves	3
2	Postmedian fascia of forewing is angled at M1; male genitalia with broad uncus, valve with a large harpe	Ptilophora nohirae
–	Postmedian fascia of is not angled at M1; uncus of male genitalia narrow, valve with a small triangular harpe	Ptilophora rufula
3	Color of forewing distal to postmedian fascia darker than basal and median areas of forewing; postmedian fascia distinct whitish, uniform from costal margin to inner margin	Ptilophora ala
–	Color of forewing distal to postmedian fascia similar to basal and median areas of forewing; postmedian fascia enlarged into white wedge-shaped spot at costal margin	4
4	Forewing pale reddish brown, with two distinct rather straight whitish fasciae	Ptilophora jezoensis
–	Forewing reddish brown to chestnut brown, basal fascia indistinct	5
5	Ground color of forewing uniformly bright reddish brown; a dusting of pale blue-gray scales in median and terminal areas of forewing; postmedian fascia white in costal area; frons near base of antennae with whitish hairs; thorax with similar long hairs at base of forewing; uncus with a triangular ventral-middle process, lateral processes serrated; valve with a triangular ventral process subapically	Ptilophora nanlingensis sp. n.
–	Ground color of forewing chestnut brown; postmedian fascia yellowish brown in costal area; frons, thorax, and abdomen with yellowish-brown hairs; uncus with blunt ventral-middle process, lateral processes with smooth margins; valva with a rounded ventral process subapically	Ptilophora horieaurea

## Taxonomy

### Ptilophora ala

Schintlmeister & Fang, 2001

Ptilophora jezoensis ala Schintlmeister and Fang 2001: 88; Kishida and Kobayashi 2002: 87; Wu and Fang 2003: 650.
Ptilophora fuscior Kishida and Kobayashi 2002: 87 [synonymised by Schlintlmeister, 2008].
Ptilophora ala ; Schintlmeister 2008: 323 [raised to species].

#### Notes.

This species can be distinguished by the postmedian fascia of the forewing, which is distinctly whitish, and the darker shading beyond the postmedian fascia.

#### Distribution:

China (Shaanxi and Sichuan Provinces).

### Ptilophora horieaurea

Kishida & Kobayashi, 2002

Ptilophora horieaurea Kishida and Kobayashi 2002: 89.

#### Notes.

This species can be distinguished by external characters: frons, thorax, and abdomen covered with yellowish-brown hairs, the postmedian fascia is covered with yellowish brown in the posterior and costal areas.

#### Distribution:

China (Sichuan Province).

### Ptilophora nanlingensis sp. n.

urn:lsid:zoobank.org:act:DA4A2641-4B7C-4102-BAE7-28578EB7E833

Figs 1 6 

#### Diagnosis.

The new species belongs to the Ptilophora jezoensis species group based on the wing pattern and male genital structure: forewing with distinct, whitish postmedian fascia, costa inflated and angulated apically. In the wing pattern, it seems to be closely related to Ptilophora horieaurea, whereas the male genital structures seem closer to those of Ptilophora jezoensis, but the two species can be distinguished from each other by the following characters: forewing bright reddish brown in Ptilophora nanlingensis, chestnut brown in Ptilophora horieaurea; uncus three-dimentional, and the costa of the valve with a rounded subapical ventral process in Ptilophora jezoensis, uncus three-dimensional, bifurcated apically, with serrated edges, and costa of valve rounded with apex inflated and with pointed subapical ventral process in Ptilophora nanlingensis (see Figs 3-5).

**Figures 1–2.**
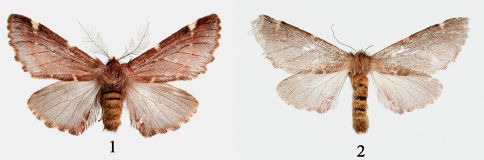
Adults of Ptilophora nanlingensis sp. n. **1** male adult **2** female adult.

#### Description.

##### Male.

Forewing length 15–18 mm. Antenna plumose, about 0.5 x length of forewing, with yellowish-white hairs at base. Thorax and abdomen with dark reddish-brown hair. Forewing ground color from bright reddish brown to fuscous brown; chocolate toward costal and posterior margins, fuscous in terminal area; antimedian fascia visible, straighter than postmedian fascia; postmedian fascia distinct, whitish, slightly convex at vein M1, forming a triangular whitish spot at costa, brighter at veins; outer margin with ground color covered with pale blue-gray scales; cilia dark brown. Hindwing fuscous; postmedial fascia absent, a whitish spot near tornus, cilia from apex to middle of outer margin yellowish brown, fuscous brown toward tornus.

##### Female.

Similar to male except forewing length 17 mm (n=1); antenna filiform; vertex, thorax and forewing uniform fuscous brown, abdomen yellowish brown.

##### Male genitalia.

Uncus three-dimensional, bifurcated apically, with serrated edges; a long triangular ventral process derive from middle of branches, smooth ventrally, slightly serrated dorsally. Socii heavily sclerotized with two horn-shaped processes. Tegumen short and broad. Valva broad with a large triangular subapical process ventrally; costa rounded with apex inflated, cucullus quadrangular; sacculus with a triangular central process, with dense setae from middle to apex. Aedeagus slender, slightly curved dorsally near caudal end, carina penis serrated dorsally.

##### Female genitalia.

Papilla analis rather broad, covered with thin hairs; the apophysis anterior shorter than apophysis posterior; ostium wide, well sclerotized; ductus bursae very lightly sclerotized, inflated mesially; corpus bursae small, membranous.

**Figures 3–6.**
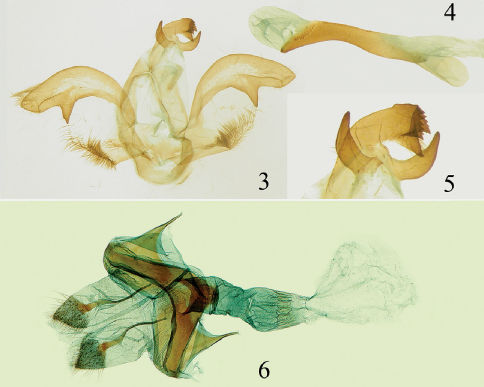
Genital structure of Ptilophora nanlingensis sp. n. **3** valva **4** aedeagus **5** uncus **6** female genitalia.

#### Holotype.

Male, Nanling, Shaoguan, Guangdong, China, 15.XII.2008, Hou-Shuai Wang leg.. Deposited in the Department of Entomology, South China Agricultural University, Guangzhou, P. R. China.

#### Paratypes.

1 female, Nanling, Shaoguan, Guangdong, China, 21.IX.2006, Min Wang leg.; 3 males, Nanling, Shaoguan, Guangdong, China, 2.XII.2007, Liu-Sheng Chen leg.; 3 males, 3.XII.2008, Liu-Sheng Chen and Hou-Shuai Wang leg., Nanling, Shaoguan, Guangdong, China; 1 male, 10.XII.2009, Hou-Shuai Wang leg., Nanling, Shaoguan, Guangdong, China. Deposited in the Department of Entomology, South China Agricultural University, Guangzhou, P. R. China. 2 males, 1 female, Yuecheng Ling, Guangxi, China, 1800 m, 26°06'N; 110°54'E, 5–8.XII.2007, Viktor Siniaev leg. Deposited in coll. A. Schintlmeister, Dresden.

#### Etymology.

The specific name is derived from the type locality: Nanling Nature Reserve, Shaoguan City, Guangdong Province.

#### Bionomics.

Bionomics. The moths were collected at light near 10°C the late autumn.

#### Remarks.

The adults of Ptilophora emerge late autumn, some species even flying at temperatures near 0°C (Kishida and Kobayashi 2002). However, adult of Ptilophora nanlingensis fly at slightly higher temperature, close to 10˚ C late autumn, which is unusual for the genus.

### Ptilophora rufula

Kobayashi, 1994

Ptilophora rufula Kobayashi 1994: 17; Wang 1996: 201; Schintlmeister 2008: 322.
Ptilophora jezoensis rufula : Shintlmeister and Fang 2001: 22 [sunk as subspecies].

#### Notes.

Kobayashi (1994) described Ptilophora rufula in Taiwan. Schinltmeister and Fang (2001) listed it as a subspecies of Ptilophora jezoensis without any explanation. Kishida and Kobayashi (2002) revalidated it as a species. It belongs to the plumigera group, based on the round valva in the male genitalia.

#### Distribution:

This species is restricted to Taiwan.

**Figure 7.**
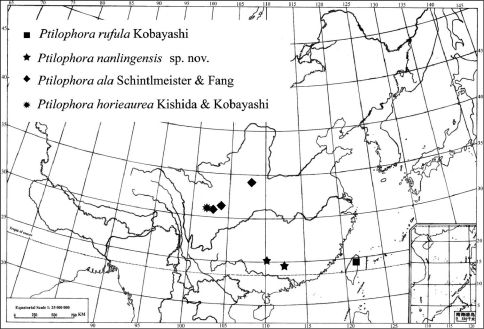
Distribution of Chinese Ptilophora species

## Supplementary Material

XML Treatment for Ptilophora ala

XML Treatment for Ptilophora horieaurea

XML Treatment for Ptilophora nanlingensis

XML Treatment for Ptilophora rufula
